# Effects of an Alpha7 Nicotinic Receptor Agonist and Stress on Spatial Memory in an Animal Model of Alzheimer's Disease

**DOI:** 10.1155/2013/952719

**Published:** 2013-08-24

**Authors:** Paloma Vicens, Diana Ribes, Luis Heredia, Margarita Torrente, José L. Domingo

**Affiliations:** ^1^Department of Psychology, Universitat Rovira i Virgili, 43007 Tarragona, Spain; ^2^Research Center in Behavioral Assessment (CRAMC), Universitat Rovira i Virgili, 43007 Tarragona, Spain; ^3^Laboratory of Toxicology and Environmental Health, IISPV, Universitat Rovira i Virgili, 43201 Reus, Spain

## Abstract

The aim of the present study was to test the effects of PNU-282987 on spatial learning and memory and hippocampal neurogenesis in both intact and chronically stressed transgenic mice. Transgenic mice with susceptibility to Alzheimer's disease (AD) under immobilization stress and not-stressed animals receiving 0 and 1 mg/kg of PNU-282987 (PNU) were evaluated in a water maze task. The effects of PNU and stress on proliferation of new cells in the hippocampus of these animals were also assessed. The latency to escape the platform was significantly higher in transgenic stressed mice compared to those in the wild stressed group, as well as in transgenic animals without PNU compared to control wild group. On retention of the task, differences emerged on stressed wild animals, PNU wild group, and stressed wild mice receiving PNU. However, no significant differences were detected on new cell proliferation. The results of the present study did not show any impact of stress in acquisition of a spatial task both in wild and transgenic mice. No clear effects of PNU on acquisition of a spatial task in transgenic mice with susceptibility to AD were detected. Although PNU and stress effects were detected on retention of the task in wild animals, no changes were noted in transgenic mice.

## 1. Introduction 

Alzheimer's Disease (AD) is characterized by a progressive loss of learning and memory processes and alterations in spatial abilities, confusion, and disorientation. One of the most known hypotheses about the etiology of AD suggests that neurodegeneration begins with an abnormal processing of amyloid precursor protein (APP), resulting in production, aggregation, and deposition of the peptide A*β*, thus facilitating the formation of senile plaques and neuronal death  [1]. Among genetic factors, in familiar AD cases, 3 autosomal dominant genes have been identified. Specifically, these encode the precursor of A*β* protein, presenilin 1, and presenilin 2, being located on chromosomes 21, 14, and 1, respectively  [2, 3]. These forms are, however, less abundant. It is expected that other genetic and/or environmental factors also contribute to the development of AD. 

Nowadays, multiple strains of transgenic mice with mutations of the peptide amyloid precursor protein (APP) have been developed. They have shown to be very useful in studying this neurodegenerative disorder. Specifically, the transgenic strain B6C3-Tg (APPswe, PSEN1De9) 85 dB/J is a double transgenic model expressing a chimera of the amyloid precursor protein (Mo/HuAPP695swe) and a mutation of the human presenilin 1 (of 9-PS1). Both mutations are associated with early onset AD. The “humanized” transgene Mo/HuAPP695swe allows mice to secrete a human A*β* peptide. These animals develop *β*-amyloid deposits in the brain at 6-7 months of age, showing a cognitive impairment at 12 months  [4, 5]. Moreover, they also exhibit impairment in different memory related behavioral tasks  [6].

Among environmental factors that may contribute to AD, stress is one of the most important. Stress is currently an unavoidable condition in our daily experiences, activating physiological systems and disregulating the body homeostasis  [7, 8]. Furthermore, stress is also considered a risk factor for a number of diseases, potentially altering neural functions that impair cognitive processes such as learning and memory through the effects of glucocorticoids levels or stress hormones (cortisol and norepinephrine) in hippocampus. Recently, it has been shown that chronic stress by immobilization accelerates cognitive decline and increases extracellular deposits of A*β* protein in a transgenic model of AD  [9]. Another recent study has shown that hippocampal cholinergic neurons in male Wistar rats become hypersensitive after chronic exposure to stress  [10].

Cholinergic neurodegeneration is considered a significant mechanism of cognitive deterioration in elderly and AD patients  [11]. Agents enhancing nicotinic cholinergic transmission have been identified as promising targets for the treatment of cognitive impairment. However, their usefulness can be limited by a number of factors, including addictive properties and adverse side effects  [12]. On the one hand, the *α*7 nicotine acetylcholine receptor (nAChR) subtype seems to mediate the protective effects of nicotine  [13–16]. On the other hand, the number of *β*4*α*2 nAChRs decreases in AD, while that of *α*7 remains largely intact, being available for binding to new therapeutic agents  [17]. Moreover, *α*7 nAChRs do not seem to be related to the addictive effects of nicotine  [18]. Preclinical studies in animals, as well as clinical studies in healthy volunteers, have shown procognitive effects of *α*7 nAChRs  [19, 20]. According to the scientific literature, with the exception of nicotine and choline, most *α*7 nAChR agonists have been partial agonists of these receptors  [20–26]. Little research has been done into selective *α*7 nAChR agonists. Only a few studies on the behavioral effects of this drug have been published. Thus, Bitner and coworkers  [11] found that A-582941, a novel *α*7 nAChR-selective agonist, enhanced the cognitive performance of monkeys, rats, and mice in behavioral studies. In turn, the *α*7 nAChR subtype was specifically shown to be involved in human memory function  [10]. Recent investigations suggest that PNU-282987 administered to rats at 1 and 3 mg/kg can reduce deficiencies in sensory auditory gating induced by amphetamine  [27]. This effect seems to be related to an increase in hippocampal GABAergic neurotransmission  [28] or to an increase of theta oscillation in the same area  [29]. PNU-282987 (PNU) has been shown to be a potent agonist of the *α*7 nAChR, having negligible interactions with other nAChR subtypes and being currently the most specific *α*7 nAChR agonist synthesized  [30]. 

As suggested by Craig and coworkers  [31], the neuronal loss or function alteration of cholinergic neurotransmission could lead to a poor ability of neural compensation to cope with secondary insults. It has been shown that different protocols of chronic stress induce alteration in neurotrophic factors  [32], oxidative damage, and alterations in antioxidant proteins  [33], which may lead to damage in hippocampus. Moreover, the initial hypothesis postulating that the generation of neurons in the postnatal hippocampal dentate gyrus is involved in the etiology and treatment efficacy of major depressive disorders has been also extended for anxiety disorders  [34]. Cognitive processes are involved in abnormal early activity reflecting hypervigilance in subcortical networks involving the amygdala and hippocampus  [35]. In fact, *α*7 subunit of the nAChR has been identified as a meaningful intermediary of nicotine's interaction with the stress axis and human disease  [36], among others. It has been shown that chronic stress produces changes such as increased nAChR *α*7 mRNA and decreased receptor binding at hippocampal formation, which suggests that nAChR *α*7 may be important for the adaptation to stress or hippocampus allostatic load  [37].

One of the most interesting plastic processes taking place in the hippocampus is neurogenesis. The concept of neurogenesis was introduced more than 40 years ago  [38]. However, it was not until recent decades that a continuous incorporation of new neurons was shown in the adult brain of rodents  [39], primates  [40], and humans  [41]. Moreover, it has been also demonstrated that new generated cells also survive over time being able to establish specific synaptic contacts  [42]. Notwithstanding, the functional role of new neurons generated in the adult brain is not well known yet. It has been shown that neurogenesis is induced by exercise and exposure to an enriched environment [42, 43], while factors such as advanced age and high levels of glucocorticoids decrease proliferation of new cells [40]. 

The present study was aimed at evaluating the possible effects of stress in precipitating the onset of deficits in spatial learning and memory in animals with susceptibility to AD. The possible therapeutic roles of PNU *α*7 agonist as well as the effects of those variables on proliferation of new cells in hippocampus were also investigated.

## 2. Materials and Methods

### 2.1. Animals and Treatment

Thirty-eight wild type and thirty-nine B6C3-Tg (APPswe, PSEN1De9) 85dB/J transgenic male mice (Charles River, Barcelona, Spain) aged 2 months were used in this study. Animals were quarantined for 10 days after shipping and housed in plastic cages in an animal room, which was maintained at a temperature of 22 ± 2°C, a relative humidity of 50 ± 10%, and on a 12 h light/dark automatic light cycle (lights on: 08.00 AM–08.00 PM). All animals were allowed free access to food (regular chow diet, Harlan, Barcelona) and tap water. Animals were randomly divided into the following 8 treatment groups.

 Wild SAL: 0.9% saline, not subjected to restraint stress, wild animals (*n* = 9).
 Wild SAL-STR: 0.9% saline, subjected to restraint stress, wild animals (*n* = 9).
 Wild PNU: PNU, not subjected to restraint stress, wild animals (*n* = 10).
 Wild PNU-STR: PNU, subjected to restraint stress, wild animals (*n* = 11).
 Tg SAL: 0.9% saline, not subjected to restraint stress, transgenic animals (*n* = 10).
 Tg SAL-STR: 0.9% saline, subjected to restraint stress, transgenic animals (*n* = 9).
 Tg PNU: PNU, not subjected to restraint stress, transgenic animals (*n* = 9).
 Tg PNU-STR: PNU, subjected to restraint stress, transgenic animals (*n* = 10).

The experimental design of the present study was approved by the Animal Care and Use Committee of the Rovira i Virgili University (Tarragona, Spain), following the “Principles of laboratory animal care.” They were carried out in accordance with the European Community Council Directive (86/609/EEC).

### 2.2. Drugs

 The *α*7 nAChR agonist PNU was purchased from Sigma-Aldrich (Barcelona). The drug was intraperitoneally administered at 1 mg/kg just before the behavioral tests. The results of a previous study suggested that only at this dose PNU-282987 significantly improved retention in the water maze [44]. PNU was dissolved in 0.9% saline, the pH being adjusted to 7. Solutions were administered at volumes of 0.1 mL/10 g of body weight. 

### 2.3. Restraint Stress

 Animals were restrained three times per day (30 min each time) during 30 days. A fixed time gap of 3 h was established between the restraint sessions. The restraint procedure consisted in placing the mice in metacrylate cylindrical holders from Letica Scientific Instruments (Panlab, Barcelona). Animals were maintained in a prone position. In previous studies, this procedure clearly caused stress in pregnant rodents [45–47]. 

### 2.4. Behavioral Tests

 To evaluate spatial learning and memory, after a month of restraining animals, were subjected to the Morris water maze test at 3 months of age. 

 The water maze consisted of a circular tank (diameter 1 m; height 60 cm), divided into four quadrants. An escape platform (diameter 10 cm) was located 1 cm below the surface of the water in the target quadrant. Animals performed 4 trials per day for 5 consecutive days. During each trial, mice were allowed 60 s to find the hidden platform and to remain on it for 30 s. If the animal failed to find the platform within this period, it was placed on it by the experimenter. The order of the three starting positions was randomized throughout the day for each mouse. To avoid proximal cues and prevent egocentric learning, an internal mobile wall was added to the maze, being the wall randomly moved between trials. This seems to increase Morris water maze sensitivity [48]. At the end of the fifth acquisition day, 4 h after the last training session, retention of the task was assessed by a probe trial, which consisted of a 60 s free swim without the escape platform. Animal performance was recorded using a video camera placed above the maze. Data were analyzed by the video tracking program EthoVision (Noldus Information Technologies, Wageningen, The Netherlands). Latency to escape the platform, distance traveled, and swimming velocity during the training sessions were measured. During the probe trials, total time spent in the target quadrant and the time spent in other quadrants were also measured in order to compare the time spent searching in the target quadrant with the average time spent in the remaining quadrants.

### 2.5. Bromodeoxyuridine Administration and Sample Collection

To evaluate hippocampus cell proliferation, two days after behavioral testing, 3 or 4 mice from each group were intraperitoneally injected with 5-bromo-2-deoxyuridine (BrdU) (Sigma, Steinheim, Germany) at 100 mg/kg/day during two consecutive days [49]. One day after the last BrdU injection, animals were deeply anaesthetized with mixed ketamine-xylazine and sacrificed by decapitation. Brains were rapidly removed from the skulls and divided coronally by free hand in two sections. Two-third posterior brain was postfixed for 4 days at 4°C in 4% paraformaldehyde. On the fifth day, brain samples were transferred to a 30% sucrose/phosphate buffered solution (PBS) for 48 h at 4°C and then snap-frozen in isopentane. Serial coronal sections, 40 *μ*m thick, were cut with a cryostat and collected according to a fractionator principle [50]. Samples were stored at −20°C in a cryoprotection buffer (40% phosphate buffer 0.1 M, 30% glycerol, and 30% ethylene glycol) for later immunohistochemical analyses.

### 2.6. Immunohistochemistry

One out of six sections was taken for biotinylated-BrdU immunostaining. Free-floating coronal sections of brain were rinsed in TBS and inactivated for endogen phosphatase activity in 0.6% H_2_O_2_-TBS. Sections were then treated for DNA denaturation incubating in 2 M HCl at 37°C and rinsed in 0.1 M sodium borate buffer. Sections were blocked in TBS-Plus containing 3% normal goat serum and 1% Triton-X in TBS for 30 min. Antibody against BrdU (Serotec, Oxford, UK) was diluted 1 : 500 in blocking buffer and incubated overnight at 4°C. Following this incubation, tissue sections were washed with TBS-Plus and incubated with secondary antibody (biotinylated anti-rat IgG, 1 : 500, Vector Laboratories, Burlingame, USA) for 2 h. After additional washes, the secondary antibody was detected using the avidin-biotin complex reaction (ABC Elite Kit). Diaminobenzidine (Vector Laboratories, Burlingame, USA) was used as chromogen. Sections were thoroughly washed, mounted, and cover-slipped.

### 2.7. Quantification of BrdU Positive Cells

Data for proliferation were obtained by using methods of unbiased stereology. The hippocampus was identified by anatomical criteria following the Mouse Brain Library Atlas [51] available at http://www.mbl.org/. The total numbers of BrdU positive cells in the granular cell layer and hilus of the bilateral entire hippocampus were exhaustively counted in serial coronal brain sections. Each section was 240 *μ*m apart from each other. The set of selected sections represents one-sixth of the whole hippocampus, being representative of the total hippocampus. Positive cells were counted by means of an optic microscopy (Olympus, CH20) through a 100× objective. Cells in the uppermost focal plane were discarded to avoid counting twice cells cut in two parts [52]. As usually, to yield an estimate of the number of BrdU positive cell numbers in the entire structure, the numbers counted were multiplied by 6 [53].

### 2.8. Statistics

Behavioral data were analyzed by means of a three-way ANOVA (Genotype × Stress × Drug). Post hoc Tukey test was used to analyze the differences between groups. Analyses for variance homogeneity were performed by means of Levene's test. A Welch's *F* and Post hoc Dunnett's T3 test were used when appropriated. One-way ANOVA for repeated measures was used to analyze spatial learning during the acquisition of the water maze. To analyze the differences between groups, a Bonferroni-adjusted pairwise comparison was used when necessary. Student's *t*-test was used for searching differences between transgenic mice and their respective wild type control groups. Nonparametrical analysis for cell proliferation was used. Statistical significance was set at *P* < 0.05.

## 3. Results

**Figure 1:**
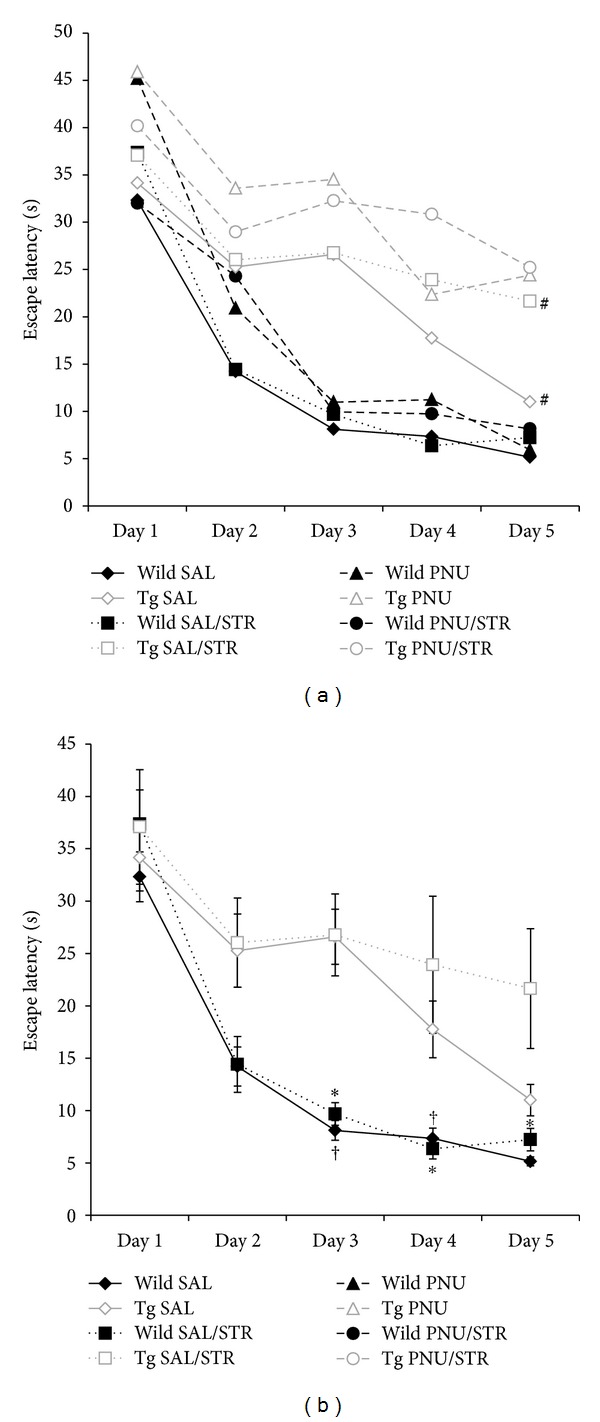
Acquisition of the Morris water maze. (a) Escape latencies in each group. A hash symbol indicates significant differences compared to their corresponding wild type groups at *P* < 0.05. (b) In order to better show differences between groups, (b) only shows escape latencies in wild type and B6C3-Tg 0.9% saline groups. Data are expressed as mean values ± SEM. An asterisk indicates significant differences between stressed wild and stressed transgenic mice at *P* < 0.05. A cross indicates significant differences between wild and transgenic saline groups.

 ANOVA for repeated measures indicated an overall effect on Group (*F*(7,68) = 6.162; *P* < 0.001), Day (*F*(4,65) = 70.509; *P* < 0.001), Day × Group (*F*(28, 272) = 2.776; *P* < 0.001), and Day × Genotype (*F*(4,65) = 15.383; *P* < 0.001) during acquisition of the water maze. Post hoc Tukey test showed that the latency to escape the platform was significantly higher in stressed and nonstressed transgenic mice (*P* = 0.038 and *P* = 0.015), in comparison to the corresponding wild mice groups (Figure 1(a)). These differences were noted on days 3 (*P* < 0.001), 4 (*P* = 0.013), and 5 (*P* = 0.044) on stressed animals but only on days 3 (*P* < 0.001) and 4 (*P* = 0.013) in control animals (Figure 1(b)). There was no significant effect of chronic stress on latency to escape the platform, in both wild type and transgenic mice. Moreover, there was no significant effect of drug treatment on the latency to escape the platform in any group. No significant differences in the distance traveled through the acquisition days were found (data not shown).

**Figure 2:**
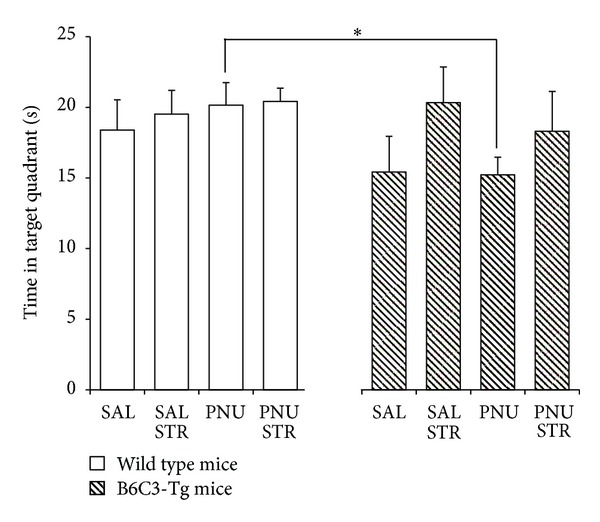
Time spent in the target quadrant 4 h after the last training session for wild type and B6C3-Tg mice. Data are expressed as mean values ± SEM. An asterisk indicates significant differences between groups at *P* < 0.05.

Retention of the water maze was evaluated by a probe trial performed 4 h after the last acquisition trial. Results showed an interaction effect between Genotype × Stress × Drug (*F*(3,13) = 4.679; *P* = 0.020). A significant difference between wild and transgenic mice receiving PNU (*t* = 2.088, *fd* = 36, *P* = 0.044) was observed on the time spent in the target quadrant (Figure 2).

**Figure 3:**
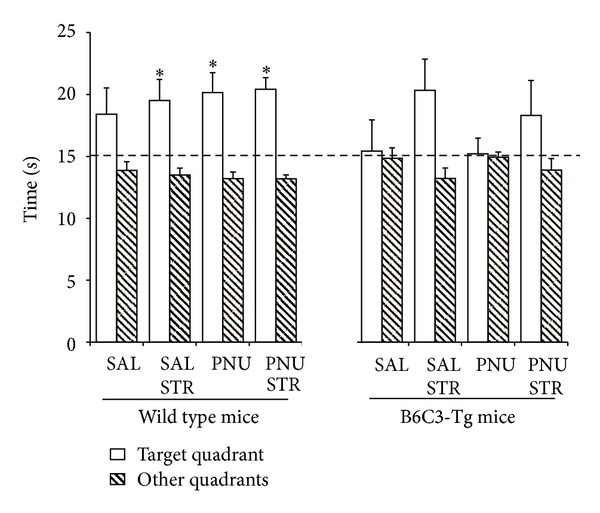
Time spent in the target quadrant in relation to other quadrants 4 h after the last training session for wild type and B6C3-Tg mice. Data are expressed as mean values ± SEM. An asterisk indicates significant differences at *P* < 0.05.

In order to better analyze differences on retention, we compared time spent in the target quadrant with the mean time spent in the other three quadrants. Differences emerged on stressed wild animals (*t* = 2.678, *fd* = 9, *P* = 0.025), PNU wild group (*t* = 3.269, *fd* = 8, *P* = 0.011), and stressed wild mice given PNU (*t* = 5.782, *fd* = 8, *P* < 0.001) (Figure 3).

No significant differences were detected on new cell proliferation (Table 1), while no correlations were noted between the number of BdrU positive cells and performance in the probe trial.

## 4. Discussion

Since cholinergic neurodegeneration has been related to cognitive deterioration in AD [11], agents enhancing nicotinic cholinergic transmission have been identified as promising targets for the treatment of cognitive impairment [54]. It has been demonstrated that chronic stress accelerates cognitive decline and increases extracellular deposits of A*β* protein in a transgenic model of AD [9]. The aim of the present investigation was to evaluate in a transgenic model of AD the possible precipitating effects of stress in the onset of cognitive deterioration in AD. The therapeutic role of PNU-282987, an *α*7 cholinergic agonist, was evaluated. The effects of stress and PNU on the proliferation of new cells in hippocampus of animals with susceptibility to AD were also assessed. 

The results indicated that although all animals learned the water maze, differences in the learning curve were noted. Although transgenic mice acquired more slowly the task, they reached similar levels as wild mice. Significant differences only emerged at the end of the learning period between wild and transgenic stressed animals, indicating a differential effect of stress depending on the genetic characteristics of the mice. It is important to consider that the current results showed a deficit in learning process in transgenic control mice compared to wild animals given 0.9% saline only (Figure 1(a)). Stress condition did not show any effect on acquisition in both wild and transgenic mice. Our data showed a possible difference in the learning basal line between transgenic and wild mice, which is probably related to the genetic characteristics of the groups. However, it is important to note that this difference was eliminated by PNU administration. No differences between animals with different AD vulnerability receiving PNU, with or without stress, were detected. These results suggest that PNU is able to improve acquisition in transgenic mice. In relation to this, it has been shown that PNU could reverse spatial deficits evaluated by a 12-arm radial maze test in rats at 3 mg/kg [55]. Moreover, although no effect on acquisition was detected in C57BL/6J, a better retention was noted after administration of 1 mg/kg of PNU [44].

With regard to spatial memory, no significant differences were observed between any experimental condition, neither in wild mice, nor in transgenic mice. Notwithstanding, a significant difference was detected between wild and transgenic mice receiving PNU. In order to better understand this difference, another analysis was performed to compare the time spent in the target quadrant with the mean time spent in the other three quadrants [49, 56]. Stress condition only, as well as PNU administration, with or without immobilization stress, improved retention of the task in wild mice only. PNU and stress had significant effects on memory on wild type animals improving their performance. However, the results did not show additive effects. Previous studies have demonstrated that moderate stress could enhance spatial memory tasks in rats [57] and mice [58].

No significant differences in new cell proliferation were found, which indicated no effects of stress on this neurogenesis process. In the scientific literature, multiple protocols to induce stress to mice may be found [59]. However, there are only few available studies using restrained stress in transgenic mice. Regarding this, in a recent study, Tg (APPswe/PS1dE) mice were subjected to 2 h per day of restraint stress during 16 consecutive days (32 h in total), showing oxidative stress increase and mitochondrial dysfunction [60]. Another recent investigation demonstrated that 6 h/day of restraint for 3 weeks excluding weekends (90 h in total) and also for 15 weeks (450 h) could efficiently induce typical physical stress symptoms in a transgenic mice model and increase hippocampal neurodegeneration [61]. In the present study, a similar number of hours were used, noting similar effects. However, this must be corroborated in further investigations, because no effects on neurogenesis were detected, while in contrast, other authors have shown clear effects of stress on new cell proliferation [62]. Moreover, we failed to find PNU or genotype effects on new cell proliferation. This could be related to the fact that transgenic mice were too young, and cell proliferation was not reduced by age, as usual. In fact, these animals have similar proliferation levels as wild type mice. It has been reported that although stress could disrupt spatial working memory both in adult an aged mice, aged animals are more sensitive [63]. 

In summary, the current data suggest possible effects of PNU on acquisition of a spatial task in transgenic mice, which should be more accurately evaluated. In this sense, it should be interesting to test the effects of an *α*7 nAChR antagonist. In turn, PNU and stress effects were detected on retention of the task in wild type animals, but no changes were detected in transgenic mice. To better understand interactions between environmental and genetic factors in AD, further studies are clearly necessary. According to the results of the present study, a longer PNU administration and/or older animals could be used for determining whether stress and PNU effects are modified by age.

## Figures and Tables

**Table 1:** Total number of BrdU positive cells in wild type and B6C3-Tg mice.

	SAL	SAL/STR	PNU	PNU/STR
Wild type mice	177.0 ± 53.81	125.3 ± 26.13	413.7 ± 122.67	286.8 ± 96.32
B6C3-Tg mice	115.7 ± 25.74	215.0 ± 22.30	295.0 ± 30.40	115.7 ± 12.30

Data are expressed as group means ± SEM.
